# A study on the evaluation method and recent clinical efficacy of bevacizumab on the treatment of radiation cerebral necrosis

**DOI:** 10.1038/srep24364

**Published:** 2016-04-12

**Authors:** Hongqing Zhuang, Xiangkun Yuan, Yi Zheng, Xubin Li, Joe Y. Chang, Junjie Wang, Xiaoguang Wang, Zhiyong Yuan, Ping Wang

**Affiliations:** 1 Department of Radiotherapy, Tianjin Medical University Cancer Institute and Hospital, National Clinical Research Center for Cancer, Tianjin Key Laboratory of Cancer Prevention and Therapy, and Tianjin Lung Cancer Center, Tianjin, China; 2 Department of Radiotherapy, Hebei Province Cangzhou Hospital of integrated traditional and western medicine (Cangzhou No. 2 Hospital), Hebei, China; 3 Daqing Oilfield General Hospital, Heilongjiang, China; 4 Department of Radiation Oncology, Division of Radiation Oncology, The University of Texas MD Anderson Cancer Center, Houston, TX, USA; 5 Department of Radiotherapy, Peking University 3rd Hospital, Beijing, China

## Abstract

In order to investigate the efficacy of bevacizumab on the treatment of radiation cerebral necrosis, patients who were diagnosed with radiation cerebral necrosis by imaging after stereotactic radiotherapy were collected. Bevacizumab was applied at a dose of 5 mg/kg once every three weeks at least three times. The changes in cerebral necrosis symptoms before and after treatment, the cerebral edema volume, the cerebral necrosis volume, and the changes in magnetic resonance imaging (MRI) strengthening phase signals of cerebral necrosis were used as the first observation point. The side effects of bevacizumab were used as the second observation point. Total of 14 radiation cerebral necrosis patients were treated with bevacizumab between June 2011 and February 2013 were collected. There were 12 symptomatic patients, of whom 10 patients (83.3%) had reduced symptoms. The edema index grades of nine patients (64.29%) improved. The cerebral necrosis volumes of 13 patients (92.86%) decreased. The T1 phase signal strengths of the intracranial enhanced MRIs of 12 patients (85.71%) significantly decreased. The clinical side effects of bevacizumab were mild. In conclusion, Preliminary results showed that treatment of radiation cerebral necrosis using bevacizumab was safe and effective. This treatment measure is worthy of further study.

Radiation cerebral necrosis is a common complication after stereotactic radiotherapy of intracranial tumors. There are no treatment measures of this disease with significant long-term efficacy^1^,^2^,^3^,^4^. One of the important reasons for radiation cerebral necrosis is the vascular mechanism^5^,^6^. As an important anti-angiogenesis drug^7^,^8^,^9^, bevacizumab is a potential candidate for the treatment of radiation cerebral necrosis. This study summarized the preliminary efficacy of treatment of patients with radiation cerebral necrosis after stereotactic radiotherapy using bevacizumab in the Department of Radiology of the Tianjin Medical University Cancer Institute and Hospital to provide preliminary references for treatment of radiation cerebral necrosis.

## Materials and Methods

### Clinical information

This study was approved by the Tianjin Ethics Committee and was performed under its supervision. All experiments conformed to the ethics requirements, and all patients signed informed consent forms. The inclusion criteria of patients were patients with primary or metastatic intracranial lesions and had histories of cyberknife stereotactic radiotherapy for brain lesions and patients who were diagnosed with radiation cerebral necrosis by imaging or pathology. The data from a total of 14 patients (six males and eight females) who had radiation cerebral necrosis after stereotactic radiotherapy of primary or metastatic intracranial tumors and received bevacizumab between June 2011 and February 2013 were collected. The ages ranged between 31 and 70 years, and the median age was 53 years. There were zero cases of primary intracranial tumors and 14 cases of metastatic tumors, of which there were 11 cases of lung cancer with brain metastasis, one case of breast cancer with brain metastasis, one case of gastric cancer with brain metastasis, and one case of lymphoma with intracranial metastasis. Four patients had received whole-brain radiotherapy in the past. Stereotactic radiotherapy was performed using 50–82% isodose line (median 75%), 1400–4000 cGy dose (median 2600 cGy), 5980–9880 cGy biologic equivalent dose (median 7590 cGy), and 1–5 segmentations (median two events). Bevacizumab treatment was conducted for 3–10 cycles (median treatment three cycles) (Table 1).

### Diagnosis of radiation necrosis

The gold standard for the diagnosis of radiation cerebral necrosis is pathological diagnosis; however, there have been many issues in clinical practice^10^,^11^,^12^. First, the locations of many intracranial tumors in stereotactic radiotherapy were close to the cranial base or in important function areas; therefore, surgical resection or stereotactic puncture could not be performed to obtain pathological diagnosis. Next, patients had very low intention for puncture after stereotactic radiotherapy. In addition, even if the stereotactic puncture was performed, the results might not completely represent the overall tissue pathology. For patients who received palliative treatment after multiple intracranial metastases, it was almost impossible to persuade patients to undergo craniotomy to confirm cerebral necrosis; furthermore, craniotomy in patients receiving palliative treatment for brain metastasis contradicted the clinical treatment purpose of prolonging survival and increasing quality of life. Therefore, although pathological diagnosis after surgery is the gold standard for radiation cerebral necrosis, it could not be achieved in clinical works. Thus, comprehensive imaging measures are the most practical and have been the most commonly applied diagnosis methods in our clinical works Therefore comprehensive imaging is the most realistic and most frequently used method in the diagnosis of brain radiation necrosis^13^,^14^,^15^,^16^,^17^. This study made a diagnosis by looking at patients’ medical history, signs and symptoms, along with results of various imaging approaches such as MRI, nuclear magnetic resonance spectroscopy, and PET-CT in this study. MRI scan and resonance spectroscopy were conducted at the beginning first, and PET-CT further introduced if the diagnosis cannot be confirmed. If still doubtful, the diagnosis should be further verified by using PET-CT. Briefly, Most brain necrosis showed irregular shape in MRI with hypointense on T1WI and hyperintense on T2WI. Moreover, liquefaction necrosis often appeared represented with lower signal intensity on T1WI and higher signal intensity on T2WI. After administration of Gd-DTPA, irregularly enhanced signal without enhanced nodular was obtained in the lesions center which was enhanced irregularly without enhanced nodular, while a large area of edema belt in T1 and T2 signal without enhancement after the administration of Gd-DTPA. A around the lesions were a large area of edema belt in T1 signal and T2 signal and no enhanced. In MRS, Cho, Cr and NAA in brain radiation necrosis lesions content levels were reduced than normal brain tissue. Cho/Cr(>1.2), Cho/NAA(>0.7) increased, and NAA/Cr radio were decreased (<1.8). In PET, the metabolic rate of brain radiation necrosis was lower than the metabolic rate that of normal brain tissue(SUV <2.5); in the corresponding region, resulting in decreased uptake of FDG and defected radioactive imaging in the corresponding region defected with decreased uptake of FDG^19^. The diagnosis was all ultimately determined by 3 independent investigators suggested by imaging. The diagnosis of brain necrosis should be finally confirmed by 3 independent doctors. If a patient had severe symptoms but also indications for surgery, the lesion could be resected and the diagnosis confirmed by histology. In this study, there were 12 patients with brain necrosis, with 1 confirmed by pathology and 11 confirmed by imaging.

### Bevacizumab treatment

The dose of bevacizumab was 5 mg/kg^18^, which was applied once every three to four weeks. Anti-allergy treatment with diphenhydramine or hormones was performed before bevacizumab treatment. The first medication time for bevacizumab was more than 90 min; afterwards, the medication time of each treatment was more than 60 min. During the medication administration, patients were monitored using electrocardiogram to closely observe their reactions to the medication.

### Evaluation criteria before and after treatment

**Figure 1:**
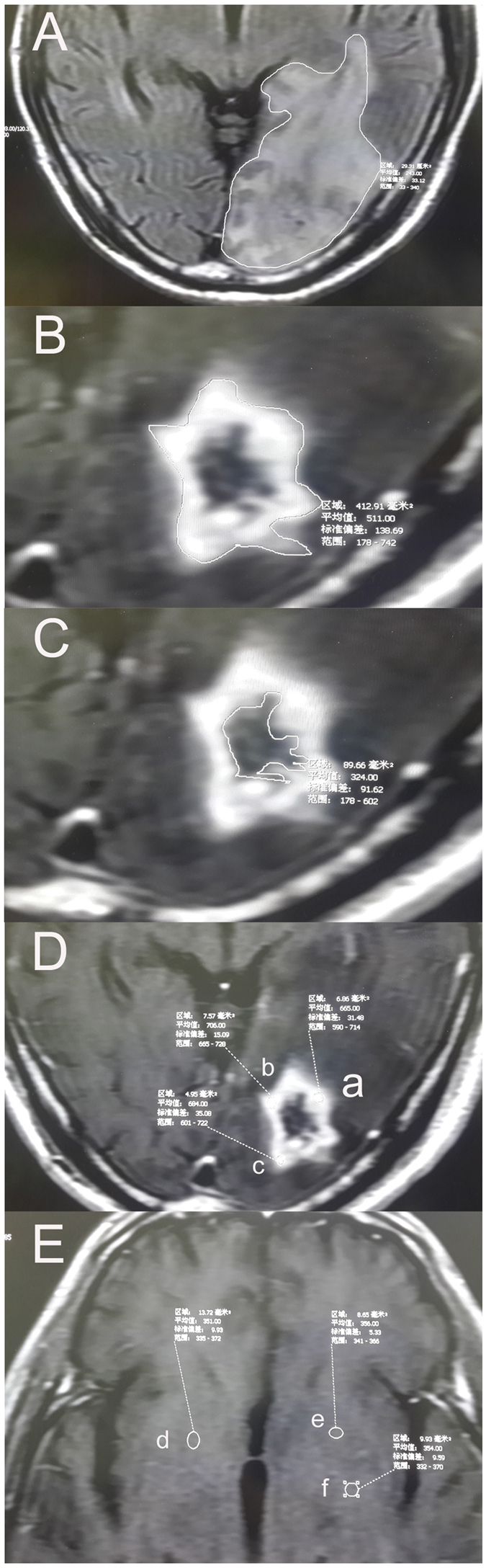
The evaluation methods of radiation brain necrosis. (**A**) The area of the edema at one layer of the MRI image. (**B**) The outer edge of radiation necrosis of the brain. (**C**) The inner edge of radiation necrosis of the brain. (**D**) Determination of MRI signal in brain necrosis area. (**E**) Determination of MRI signal in cerebral white matter area at the same level. The volume was calculated using the product of the area of the edema region and the slice thickness. The edema index (EI) was used for evaluation. The EI = Volume A/Volume B. The volume of cerebral necrosis was calculated using the subtraction of the inner diameter volume from the outer diameter volume of the enhancement region of cerebral necrosis(Volume B subtract Volume C). The changes in information in the region of cerebral necrosis used the measurement of the T1 strengthening phase. Three areas in the strengthening region of cerebral necrosis were selected to measure the signal values and to calculate the mean value; in addition, the value was compared with the white matter signal value of the same MRI to eliminate the influences of different strengthening degrees; this ratio was used to measure the changes in signals in the regions of cerebral necrosis before and after treatment.

One month after treatment with three cycles of bevacizumab, patients received routine intracranial magnetic resonance imaging (MRI) examinations. Afterwards, MRI examinations were performed every two to three months for one year. MRI examinations were then performed based on the conditions of the patients for durations of no longer than six months. If there were intracranial symptoms, an examination was performed immediately. The changes in patient symptoms after bevacizumab treatment were evaluated according to the Common Toxicity Criteria 4.0 (CTC4.0) standard. The edema range was measured at the fast-recovery fast spin-echo-T2-weighted (FRFSE-T2WI) phase and drawled on MR via free-hand manner. The volume was calculated using the product of the area of the edema region and the slice thickness. The edema index (EI) was used for evaluation. The EI = Volume (edema + necorosis)/V (necorosis). In Fig. 1, The EI = Volume A/Volume B. EI = 1 was regarded as no edema, 1 < EI ≤ 1.5 as mild edema, 1.5 < EI ≤ 3 as moderate edema and EI > 3 as severe edema^20^. The T1 phase of contrast MRI was used to measure the changes in volumes of cerebral necrosis. And the fields of necrosis were drawled on MR via free-hand manner. Considered the targets of bevacizumab, the volume of cerebral necrosis were calculated using the subtraction of the inner diameter volume from the outer diameter volume of the enhancement region of cerebral necrosis (Volume B subtracts Volume C). The measurement of volume was conducted using the same method as described above. The changes in signals in the region of cerebral necrosis used the measurement of the T1 strengthening phase. Three areas in the strengthening region of cerebral necrosis were selected to measure the signal values and to calculate the mean value; in addition, the value was compared with the white matter signal value of the same MRI to eliminate the influences of different strengthening degrees; this ratio was used to measure the changes in signals in the regions of cerebral necrosis before and after treatment (Fig. 1).

### Statistical methods

SPSS 17.0 software was used. *P* < 0.05 was the standard of statistical significance. The differences of changes in symptoms, edema index, cerebral necrosis volume, and signals in cerebral necrosis regions before and after bevacizumab treatment were analyzed using the t test.

## Results

### Side effects of bevacizumab treatment

One patient had mild allergy, which improved after symptomatic treatment. One patient had one incidence of increased blood pressure, which was relieved automatically. There were no reactions of grade 2 or above. There were no reactions to bevacizumab application, such as skin rash, fatigue, proteinuria, thrombosis, hemorrhage, gastrointestinal perforation, delayed wound healing, reversible posterior leukoencephalopathy syndrome, and congestive heart failure.

### Changes in symptoms

Among these 14 patients, 12 patients had increased intracranial pressure-induced mental disorders, including headache, dizziness, nausea, vomiting, and memory loss and vision disorders, such as visual field defect before treatment. After bevacizumab treatment, 10 patients (83.3%) had reduced symptom grades. The specific conditions are described in Table 2. The paired t test result showed that t = 5.657 and *P* = 0.000 (Table 2).

### Changes in edema degrees

**Figure 2:**
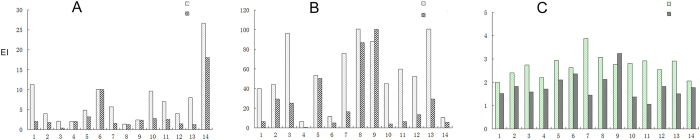
The treatment effect of bevacizumab. (**A**) the edema index (EI). (**B**) The volume of cerebral necrosis. (**C**) The changes in information in the region of cerebral necrosis used the measurement of the T1 strengthening phase.

The edema index was calculated according to the degrees of patient edema; in addition, the changes in edema before and after treatment were compared. The edema index grades of nine patients (64.29%) improved (Fig. 2A). The edema indices before and after treatment were significantly different (t = 3.946, *P* = 0.002).

### Changes in volume of cerebral necrosis

After bevacizumab treatment, the volumes of cerebral necrosis of 13 patients among the 14 patients (92.86%) were significantly reduced. Examinations revealed that the volumes of cerebral necrosis before and after treatment were significantly different (t = 3.952, *P* = 0.002, Fig. 2B).

### Changes in MRI signals of cerebral necrosis

Through the measurement of the relative strength of the T1 phase signals in intracranial enhanced MRI, the changes in blood supply in the lesions of cerebral necrosis before and after bevacizumab treatment were measured. The signals in 12 patients (85.71%) significantly decreased, and the signals of cerebral necrosis before and after treatment were significantly different (t = 4.507, *P* = 0.001, Fig. 2C).

## Discussion

Preliminary results indicated that treatment of radiation cerebral necrosis with bevacizumab was safe and effective.

The mechanisms underlying the treatment of radiation cerebral necrosis with bevacizumab should be discussed in terms of the mechanisms of the occurrence of radiation cerebral necrosis and angiogenesis inhibition by bevacizumab. The mechanism of vascular injury plays an important role in the developmental process of radiation cerebral necrosis. Radiation functions on blood vessels to cause fibrinoid changes in vascular endothelial cells, thus inducing tissue hypoxia and necrosis. During this process, large amounts of cytokines (such as vascular endothelial growth factor–VEGF) that function on blood vessels are released. These factors gradually cause blood-brain barrier dysfunction and cerebral edema, thus affecting corresponding neurological functions^21^. In addition, radiation can induce astrocyte injury to further cause VEGF release, thus resulting in blood brain barrier injury and further aggravation of cerebral edema. Therefore, application of bevacizumab to block VEFG release to target capillaries, to decrease vascular permeability, to reduce inflammatory factors carried by plasma or plasma flow to reach extracellular regions through capillary endothelial cells, and to reduce blood-brain barrier injury and brain tissue edema might become a more reasonable regimen for the prevention and treatment of radiation cerebral necrosis. In radiation cerebral necrosis animal models, VEGF is expressed at high levels, which also further confirmed the existence of such a mechanism. Thus, the mechanisms underlying the radiation cerebral necrosis and the anti-angiogenesis functions of bevacizumab could have treatment effects on radiation cerebral necrosis; results in this study also confirmed this mechanism^22^,^23^.

Compared to previous studies^24^,^25^,^26^,^27^, radiation cerebral necrosis is a common complication in radiotherapy of intracranial tumors. For a long time, the condition was treated symptomatically using hormones or measures such as hyperbaric oxygen; however, the efficacy of this treatment regimen was not satisfactory. Surgical resection was influenced by the location of the appearance of cerebral necrosis and resulted in more severe patient trauma. Evaluating the treatment of radiation cerebral necrosis with bevacizumab provided an effective and new exploration of treatment measures for clinical practice; it was truly a new way of thinking for the treatment of radiation cerebral necrosis.

Overall, through the investigation of the preliminary efficacy of bevacizumab in the treatment of radiation cerebral necrosis after stereotactic radiotherapy, this study provided a new mindset regarding the treatment of radiation cerebral necrosis. Although the patient cases in this study were fewer and the clinical observations were preliminary, this study provided a new method for the treatment of radiation cerebral necrosis complications after stereotactic radiotherapy of intracranial tumors, which benefited the clinical practice of current stereotactic radiotherapy of intracranial tumors. With the further accumulation of clinical data and further summarization of clinical experiences, we believe that there will be more extensive clinical application prospects for the treatment of radiation cerebral necrosis using bevacizumab.

## Additional Information

**How to cite this article**: Zhuang, H. *et al.* A study on the evaluation method and recent clinical efficacy of bevacizumab on the treatment of radiation cerebral necrosis. *Sci. Rep.* **6**, 24364; doi: 10.1038/srep24364 (2016).

## Figures and Tables

**Table 1:** Baseline of patient and treatment characteristics.

Characteristic	Value
Cases	14
Gender(cases/percent)	
male	6/43
Female	8/57
Age(year)	
Range	31–70
Media	53
Primary or metastases (cases/percent)	
Primary	0
Metastases	14
Primary site (cases/percent)	
Lung	11/78.6
Breast	1/7.1
Lymphoma	1/7.1
Gastric cancer	1/7.1
WBRT (cases/percent)	
Yes	4/28.6
No	10/71.4
Dose(cGy)	
Range	1400–4000
Media	2600
Dose line(%)	
Range	50–82
Media	75
Fractions	
Range	1–5
Media	2
BED (cGy)	
Range	5980–9880
Media	7590
Frequency of bevacizumab	
Range	3–10
Media	3

**Table 2:** The Symptom changes of the patients before and after treatment.

Cases	Main symptom	Grade of symptom before treatment	Grade of symptom after treatment
Case1	headache	2	0
Case2	papilledema	2	0
Case3	headache	2	1
Case4	none	NA	NA
Case5	headache	2	2
	memory impairment	2	2
	Vomit	1	1
Case6	none	NA	NA
Case7	dizziness	2	0
Case8	dizziness	2	1
	headache	2	1
Case9	headache	2	2
	nausea	2	2
Case10	somnolence	2	0
Case11	headache	3	0
Case12	headache	3	1
	nausea	2	0
Case13	dizziness	2	0
Case14	headache	1	0

The result of Paired Sample T test: t = 5.657, P = 0.000.
